# A Parametric Study for Tensile Properties of Silicone Rubber Specimen Using the Bowden-Type Silicone Printer

**DOI:** 10.3390/ma15051729

**Published:** 2022-02-25

**Authors:** Jing Angelo Gonzaga Clet, Nai-Shang Liou, Chen-Hsun Weng, Yu-Sheng Lin

**Affiliations:** 1 Department of Mechanical Engineering, Southern Taiwan University of Science and Technology, Tainan 710301, Taiwan; ma91y212@stust.edu.tw (J.A.G.C.); nliou@stust.edu.tw (N.-S.L.); 2 Medical Device Innovation Center, National Cheng Kung University, Tainan 70101, Taiwan; b88501113@gmail.com

**Keywords:** silicone printing, parametric study, taguchi method, tensile properties

## Abstract

Silicone printing can enable a lot more accessibility and customizability towards utilizing silicone in different applications, including medicine for its biocompatibility. However, challenges existed for printing in specific geometries due to the lack of guidelines and studies on the mechanical properties. To support the understanding of printing three-dimensional silicone structure having different infill patterns and gel-like material, this paper conducted a parametric study for the specimens printed using a Bowden-type silicone printer and measurements of the tensile properties. Four printing parameters of print speed, infill density, flow rate, and infill pattern, are categorized following the Taguchi L9 method, and arranged into the four-parameter-three-level orthogonal array. The signal-to-noise (S/N) ratio was calculated based on the principle of the-larger-the-better, and analysis of variance (ANOVA) was also obtained. Tensile performance was further discussed with the characterization of internal structure, using the cross-sections of the printed specimens. It was found that the change of flow rate is the most significant to the tensile stress; and for the tensile strain, infill pattern was found to be the most significant parameter. The Line infill pattern consistently presented the highest tensile stress. Agglomeration can be seen inside the printed structure, hence optimal printing parameters play an important role for complicated geometry, while ensuring the flow rate and infill density do not exceed a reasonable value. This study would serve as the guideline for printing three-dimensional silicone structures.

## 1. Introduction

Additive manufacturing technology, so-called 3D printing, has been on the rise in the recent years. In particular, 3D printing has seen commercial success in the realm of hobbyists and industrial prototyping fields, even reaching the heights of aerospace industries [1]. The usual material that 3D printing utilizes is thermoplastics, with Polylactic Acid (PLA) being the most prevalent one, due to their nature to become fluid at extreme heat and cool instantly at room temperature [2,3]. Recently, there have been studies on using various material such as silicone rubber, for additive manufacturing technology [4,5,6]. This brings additional possibilities as the biocompatible nature of silicone rubber would enable this technology to bring improvements towards the fields of medical and assistive devices.

One outcome of this is that Silicone Printing has started to have a presence in the commercial market, such as the S052 from SanDraw [7]. This printer uses a two-part silicone in barrels as the ‘filament’. It is extruded through linear movement of the stepper motor, bringing the silicone components onto connections of tubes until it reaches the nozzle. The tubes of two components converge into one tube, and this tube has an embedded mixing blade to ensure that the components are thoroughly mixed. The mechanism of this printer is similar to Material Extrusion (MEX) technology regulated in ISO/ASTM 52900, with the exception of thermal reaction bonding [8]. It primarily uses tubes as the medium for extruding silicone, as compared to the direct extrusion configurations of the studies in [4,5,6]. This Bowden configuration exists already in regular 3D printing, offering advantages in significantly reducing the weight of the moving mechanism, increasing possible print speeds without facing jitter problems. A disadvantage, however, is posed by the material traveling in the said printing tube, having extrusion problems in flexible filaments [9]. Its configuration would thus need its own analysis in order to overcome the associated challenges by the material silicone. In addition to providing commercial presence, a specific notable feature would be the material that can be extruded onto the printer with the desired 3D objects. These silicone materials have been approved in the standard of ISO-10993 [7]. This standard evaluates the biocompatibility of devices [10]. Thus, overcoming the challenges of this printer would enable rapid prototyping of biocompatible materials for a variety of applications.

It is then important to note the strategies used in silicone printing research to have an idea on how to tackle these challenges. The study in [11] makes use of simultaneous control of reaction kinetics and transient rheology. They determined time and temperature in boundary conditions for the amount of curing during extrusion and layering of the print. In their printing results of 3D models, they were able to produce overhanging and enclosed hollow structures that require minimal post-processing. The study in [12] talked about the extrusion of low viscosity inks. They made use of microfluidic print-heads for multiple ink switching and an ultraviolet exposure system for fast curing of their silicone prints. This allowed them to produce structures without needing support material. These methods are, however, limited not only in their type of silicone printer, but in the different characteristics of silicone material used by other printers. In contrast, a specialized silicone material is then studied in [13], where they reported a guide for printing highly stretchable silicones. They built a theoretical model to effectively control printing speed and accuracy. Their resulting prints include the highly stretchable silicone, complex structures, soft electronics, and artificial muscles. These are useful for their silicone printer mechanism, where piston rods extrude the silicone components into a mixing tube with orthogonally arranged curved mixing blades that then extrudes out of a nozzle. This would be similar to the previously mentioned silicone printer of SanDraw, where it enables simultaneous mixing and printing. This stabilizes rheological properties mid-print and extends printable time of the silicone component.

Another more applied example of using silicone printing would be the soft robotic fabrication study by [14], where they compare the soft robot’s performance with their molded counterparts. For their printer, they made use of mixer chambers and convective heater for ease of mixing and curing of the silicone structures. The configurations of MEX printer however differs largely from the Bowden-tube configuration of the SanDraw printer in terms of their mechanism. Thus, the resulting printed structures would contain differences in both external appearance and internal structure. 

One such challenge is the setting of parameters to achieve said additive manufacturing for this experimental material and configuration. For the regular 3D printing of PLA, studies that involve parameter setting is shown in [15], where the specimens are under water-absorption, and heat-treated conditions. They studied how layer-height, build-orientation, and raster orientation affected impact resistance. The study in [16] then shows effects of printing parameters as well as annealing operations towards the porosity and crystallinity of the structure. However, the large parameter space of 3D printing slicers makes it difficult to achieve optimal parameters, even using a different material for extrusion. Parameter optimization by [4,5] responded with problems in their aim to achieve silicone printing specifically through freeform reversible embedding (FRE). FRE involves printing the structure under an aqueous bath for support. The expert intervention system in [4] allows a more structured print with an optimized process. They started with screening the selection of parameters such as space, factor, and factor levels, and then used a hill-climbing algorithm to search the parameter space for the parametric optimization. The factors that they chose to optimize include the following: layer height, speed, filament packing density, retraction distance, solid surface thickness, tower, and comb. They evaluated the results based on print quality by assigning numeric scores, qualitative descriptions for layer fusion, infill, and stringiness. They were able to optimize the printing of a hollow cylinder, then, applied those parameters in printing 3D objects with increased structural complexity. As a response to this using another method, it is the chemical properties of the bath and ink material, along with printing settings that [5] used in their machine learning model to create optimized printing settings for the same type of silicone printing mechanism. Their algorithm estimated processing conditions that enabled them to use faster print speeds when using low-viscosity inks. These two studies created strategies for FRE, but this specialized addition of manipulating the parameters of the support bath can deviate from the typical room temperature printing setups that other 3D printers already have. 

An application of silicone printing into fabricating a meniscus implant is shown in [17]. Here, they studied how printing parameters affect dimensional accuracy and mechanical properties, reliability and failure analysis of the printed silicone, and its cytotoxicity. Their results show low cytotoxicity and excellent biocompatibility of printed silicone. They analyzed their printing parameters via regression analysis and characterized the meniscus through compressive tests. This, however, is limited to the structure of the meniscus, and there would be some needs of doing tests on established structural standards. The study also studied the compressive property of the structure, however without an understanding of the tensile property instead. The same meniscus analysis but focusing more on using the configured printer was done in [18]. The printers they used were more akin to the Bowden-tube configuration, but also used external heating elements on the nozzle and bed to assist the curing process. The focus on printing meniscus would also face the previously stated limitations. 

Experimental design of optimization is an additional method to obtain optimal processing parameters, and the Taguchi method is one of the most efficient methods. This method is used in work about additive manufacturing such as [2,3,19,20], in the efficient determination of optimum parameters in MEX technology for different outputs. The MEX study in [2] investigated the effects of extrusion temperature, layer thickness, infill percentage, and infill patterns on dimensional accuracy, repeatability of dimensions and mechanical properties. They found that a better dimensional accuracy can be obtained in lower extrusion temperatures, smaller layer thickness, lower infill percentage, and a hexagonal infill pattern. On another hand, ref. [3] chose to use infill density, layer thickness, and print speed to optimize the output tensile properties of PLA specimens. They found their optimal conditions for the best tensile properties to be 80% infill density, 40 mm/s print speed, and 0.1 mm layer thickness. These showed the reliability of using the Taguchi method. The geometrical properties of dimension and tolerance was the output optimized by [19] in their Taguchi experiment. The study in [20] on the other hand used Taguchi for a more specified MEX printing, as they optimized process parameters for fabricating lattice structures. These were more in line with the usual thermoplastic materials in 3D printing technology. The spring assistive mechanism in [21] pertained to Digital Light Process (DLP) 3D Printers. Their research used the Taguchi method to determine the factor importance of the processing parameters in their spring assisting mechanism. Taguchi helped them discover that the separation force is small when spring stiffness is small, spring loaded length is short, and spring free length is long. 

Relating to the output parameters, other than [3], tensile properties have also been the subject of optimization using Taguchi in [22,23,24]. Injection molding process parameters of carbon fiber content, holding pressure, injection speed, and barrel temperature were investigated in [22]. The advantages of Taguchi and Six Sigma approach were demonstrated in their findings that included increasing carbon fiber content improves the tensile strength. The MEX study in [23] studied the parameters of build orientation, raster orientation, nozzle diameter, extruder temperature, infill density, shell number, and extruding speed on tensile strength in PLA filaments. They found that build orientation, nozzle diameter, and infill density have a high statistical significance, and thus have high impact on the result. The Analysis of Variance (ANOVA) method assisted in verifying the Taguchi results in [21,23,24]. The Taguchi and ANOVA results, in conjunction with neural networks, were useful for [24] in finding parameters from the flux, varying time, input current, and electrode diameter that generate higher tensile strength for Tungsten Inert Gas welding.

In terms of tensile properties, they are the main criteria in characterizing silicone structures for various applications, with flexibility being its defining characteristic. They are then used for characterization in research not limited to the Taguchi method. One example related to characterizing printed silicones is in [6], which made a study in terms of their tensile and void properties. They used a different printer than the previously mentioned studies, as their silicone material is moisture-cured and is extruded through a progressive cavity pump attached to a nozzle. They varied the infill direction, and adjacent line spacing to study tensile properties and void formation. They found that internal void geometries increasing due to their mechanism operations significantly reduces the average tensile strengths of their specimens. They supported these results by using optical microscopy to determine failure points of their printed specimens. They found failures occurring in bottom surfaces of their minimal void specimens, where they attribute it to uneven curing creating cross-link density differences. These results led them to conclude that for their type of printer, it is advantageous to minimize voids between adjacent line spacings, jagged edge geometry, internal tangency voids, and surface roughness. The same type of silicone printer was also studied in [25], where biaxial tensile properties and hyperelastic material models were used to evaluate additive manufacturing of wearable biomedical devices. Their research showed that the Yeoh model is the best match to their experimentally measured stress-strain curve and their finite element analysis of force and strain. These findings are useful for printers that use a mechanism similar to a progressive cavity pump directly attached on the nozzle of the machine, but may not be true for other mechanism configurations. These results thus showed the gaps in body of literature pertaining to the additive manufacturing technology of silicone components extruded in a Bowden-tube configuration.

This paper aims to do a parametric study for the tensile properties of silicone rubber specimens printed using a Bowden-type silicone printer. The printing parameters selected in this study are the print speed, infill density, flow rate, and infill pattern. The levels of these parameters are arranged in an orthogonal array based on Taguchi L9 method. The tensile tests are conducted by following the standard of ASTM D412. The results show the tensile properties, and analysis of the signal-to-noise ratios and ANOVA significance with the parameter effects. 

## 2. Materials and Methods

### 2.1. Specifications of Printing

In this study, a silicone printer SanDraw S052 (S052, San Draw Inc., Taichung, Taiwan) is introduced for investigation. It uses two customized horizontally attached plastic barrels that hold the components of the silicone mixture (Sil50, San Draw Inc., Taichung, Taiwan) simultaneously. Its mechanism is similar to Bowden style 3D printers where the silicone barrels are connected by a tube to the nozzle. For the movement of the nozzle, two stepper motors have threads attached to them, making the X and Y axes of the printer, have a pulley system style of operations. The z-axis has two stepper motors, and these are attached to threaded rods that are screwed into the bed. The home positions of the x and y axes are indicated by limit switches. Calibration of the z-axis makes use of a sensor component that is adjusted manually using micrometer knob. There is a glass plate attached onto the bed of the silicone printer for the post baking process. These act similar to a pallet system to enable the ease of handling workpieces, for putting them onto the oven for curing, for storage, and for executing printing processes while the curing operations are being done. 

**Figure 1:**
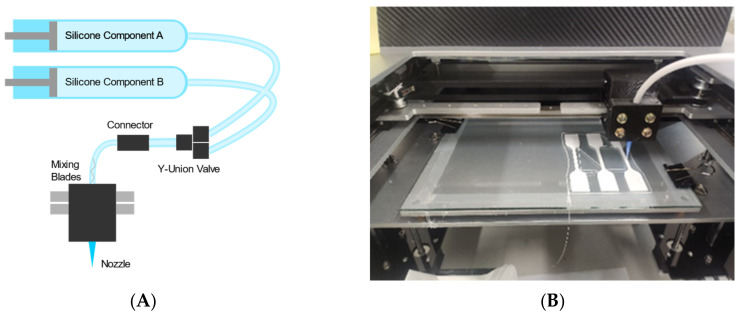
(**A**) A schematic diagram of the printing mechanism; and (**B**) a photo of printing the silicone dumbbell specimens.

Extrusion of the silicone components in the barrels is achieved through the threaded rod attached to the stepper motors. These rods push the airtight lid of the horizontally attached barrels, which in turn exerts pressure onto the silicone components, making them flow to the tubes and then onto the nozzle. Melding of the two components of silicone is achieved through the mixing blades embedded into the tube connected to the nozzle. This nozzle has an inner diameter of 0.4 mm, similar to the inner diameter of a regular 3D printer. A schematic diagram of printing mechanism of S052 and an example of printing operation can be seen in Figure 1A,B, respectively. The Bowden-tube configuration enables this printer to exhibit faster speeds compared to printers in a direct drive configuration, due to having less mass in the moving mechanism [9]. The challenge would thus be in working with the delay of extruding the material through the long tubes to the nozzle. 

The software (FAMufacture, San Draw Inc., Taichung, Taiwan) used by the silicone printer utilizes the same principles and parameters that can be seen in 3D slicer software such as Cura (version 4.13.1, Ultimaker, Utrecht, The Netherlands) [26]. The general workflow for the software is that an STL file is first loaded and positioned onto a virtual space that corresponds to the bed of the printer, having the options to move, scale, rotate, and other features of a regular 3D slicer. These STL files are then sliced in accordance to the configured print parameters. The software is also needed for the actual printing process; it sends the G-code commands to the printer firmware and then printer executes it.

### 2.2. Silicone Material

The used silicone material Sil50 (Sil50, San Draw Inc., Taichung, Taiwan) is a two-part liquid silicone rubber (LSR) and is colored opaque white. It gets its name from its hardness of shore A 50. It has a tensile strength of 1750 psi or 12.07 MPa and a tear strength of 40 kgf/cm [27]. These parameters are provided by the supplier of the company without testing the use of additive manufacturing. As previously mentioned, this material is approved under ISO-10993. This biocompatible feature of this material would thus enable it to be used for a variety of medical, assistive, or other related applications.

### 2.3. Design of Experiments

For this study, the Taguchi method of experimental design is chosen for its robustness and efficiency [28,29]. Specifically, the Taguchi L9 method is chosen that uses four parameters and three levels. Four printing parameters are chosen as the input variables, with three levels for each of those parameters for demonstrating repeatability. These parameters and levels are then arranged in an orthogonal array. For the reliable study, seven specimens are printed for each combination and undergone the same experimental testing procedure. Five of these results are averaged in the following Section 2.5 Data processing. The reason of preparing seven specimens is to avoid there being any serious random errors occurred.

**Figure 2:**
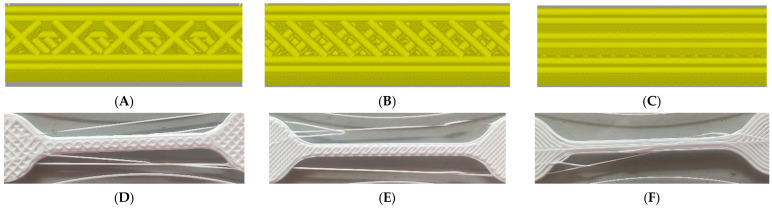
(**A**–**C**) The shapes of the sliced preview image of the (**A**) Grid, (**B**) Line, and (**C**) Concentric infill patterns used in the study. (**D**–**F**) The resulting (**D**) Grid, (**E**) Line, and (**F**) Concentric infill patterns printed by the silicone printer. These are all printed at 60 mm/s and at a 53% infill density to visually show the intended patterns.

The following parameters in the FAMufacture software are selected due to their large contribution toward the success of printing the 3D structure [26]. Printing speed dictates the general movement of the gantry, infill density is the material density of the structure printed, flow rate is the speed of extrusion of the two parts of the silicone mixture, and the infill pattern tells the arrangement of toolpaths in the inner part of the structure. The levels of the print speed chosen are 10 mm/s, 35 mm/s, and 60 mm/s. Choosing a lower speed than 10 mm/s makes the printing process more inefficient, and having a larger speed than 60 mm/s does not make it possible for the nozzle to deposit the mixed silicone properly. The middle level of 35 mm/s is chosen to be the midpoint of the two ends of the upper and lower levels. The infill density levels are 15%, 53%, and the largest possible, 90%. The lowest level is chosen as the company stated that it is the minimum possible density for the structure, and the middle one is the midpoint between 15% and 90%. The 90% limit is chosen to show the contribution of the infill pattern. The flow rate levels are 50%, 75%, and 100%. Choosing the upper and lower levels for these parameters is the same as the one in the printing speed, as having a lower level than 50% will not make the printing efficient and 100% is the maximum value where the output print can still exhibit the intended dimension and material properties. The infill patterns used are Grid, Line, and Concentric, which can be seen in Figure 2. The grid pattern stacks a grid on the layers to accommodate the infill of the structure. The line pattern shows diagonal lines on each layer, with alternating directions of diagonal lines per layer. The concentric pattern follows the shape of the dumbbell in a concentric manner. The print parameter values, and their corresponding levels are configured in the orthogonal array as shown in Table 1.

In addition to the specimens printed using the parameters of the orthogonal array, specimens printed at 100% infill and molded specimens are also made to have a baseline comparison. The 100% infill specimen is printed at a 60 mm/s speed and at a 100% flow rate. The parameters of print speed and flow rate are both optimized after doing the orthogonal array experiments, where its selection is based upon which would let it retain the specimen dumbbell shape without much deviation. The molded specimen is fabricated through creating a 3D printed mold for the shape of the dumbbell specimen using a Fused Deposition Modeling (FDM) printer and then pouring the extruded silicone in the said mold. The extruded silicone is then spread onto the mold.

The constant parameters in the experiments include the nozzle diameter of 0.4 mm, shell thickness of 0.8 mm, top and bottom thickness of 0.6 mm, and infill overlap of 15%. The function of retraction is not applicable during the printing since the gel-like silicone material is difficult to withdraw. These are printed at room temperature, ranging around 21 to 23 degrees Celsius and in a humidity of around 30–50%.

### 2.4. Tensile Tests 

**Figure 3:**
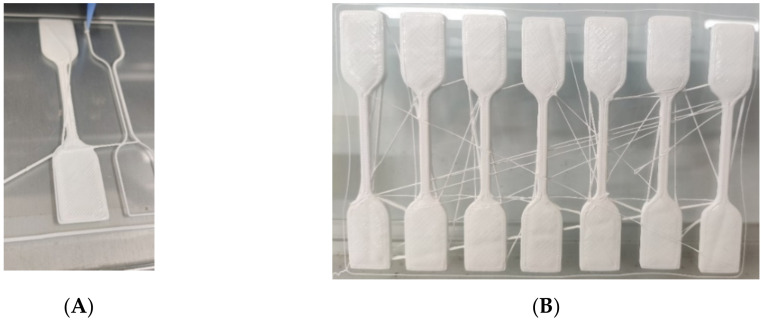
The photos of (**A**) printing the first layer of the dumbbell specimen; (**B**) the cured dumbbell specimens in one combination group.

The specimens used for this study follow the standard in ASTM D412 for type-D dumbbell shape specimens with a thickness of 3 mm [30]. The printing toolpath of the specimens is dictated by the software (FAMufacture, San Draw Inc., Taichung, Taiwan) provided by the company, which is a slicer and printer operating software that uses Cura as a base. There are three specimens printed with the same parameter combination simultaneously, with an example shown in Figure 3A, having a total of 27 specimens for nine parameter combinations. After printing, these are cured in an oven with temperature and time settings of 80 degrees Celsius for 2 h, and 130 degrees Celsius for another 2 h. These are based on the instructions, provided by the company, on post-curing the silicone printed structures. The procedure for the preparation is consistently applied in all experiments. Figure 3B shows an example of the cured specimens in one combination group. 

The tests are performed using the Instron 5865 tensile testing machine (5865, Instron, Norwood, MA, USA.) with a 5 kN Load Cell. Accordance to the standard of ASTM D412, the tests are done at a rate of 500 mm/min [30]. 

### 2.5. Data Processing

The output data are processed through the software OriginLab (OriginPro 2016, OriginLab Corporation, Massachusetts, MA, USA.), including calculation and statistical analysis. It is recorded until the specimens all break at the maximum force. Tensile tests output force and displacement data. The displacement data are then used to calculate engineering strain denoted by the equation

ε_e_ = ΔL/L_0_, (1)

where ΔL is the displacement recorded by the tensile testing machine and L_0_ is the original length of the Type D specimen based on ASTM D412 dimensions. On the other hand, engineering stress is calculated by

σ_e_ = F/A_0_, (2)

with F being the force recorded by the tensile testing machine and A_0_ the cross-sectional area of the Type-D specimen based on ASTM D412. Considering the tensile behavior of hyperelastic materials, such as silicone rubber, the area of cross section deforms relatively more during the process of elongation. Thus, the typical assumption of the cross-sectional area not changing during the deformation process is not applicable. These engineering stress-strain properties are then used to calculate true stress-strain properties, based on the instantaneous cross-section area and length [31]. The true strain is denoted by

ε_t_ = ln(1 + ε_e_), (3)

and the true stress is denoted by

σ_t_ = σ_e_ × (1+ ε_e_). (4)

The maximum values of tensile stress and tensile strain are compiled for each of the specimens. These data are then arranged according to the input printing parameter and their levels. After compilation of the maximum output values of tensile stress, and tensile strain for each specimen in the orthogonal array, the Signal-to-Noise (S/N) Ratio is calculated. This is done using the larger the better formula:

S/N Ratio (larger the better) = −10 × log(Σ(1/Y^2^)/n), (5)

where Y is the output data, and n is the number of outputs. The output data come from the repeated tests of the combination. This is done for each combination, using the data of their corresponding three specimens following the Taguchi process of selecting the optimal print parameter levels [28,29]. For the S/N ratio, a higher value indicates a more desirable variability condition, wherein this experiment used the higher-the-better characteristic [29].

The corresponding S/N ratio per parameter level is then sorted and averaged. This is done by grouping first the combinations based on one parameter. An example would be grouping engineering stress results by print speed: the combinations with 10 mm/s print speed are combined and the average of their S/N ratio in engineering stress is obtained. Similarly, the combinations with 35 mm/s and the 60 mm/s print speed would be averaged. This same operation for all parameter levels would be done for infill density, flow rate, and infill pattern. This process is then repeated for a different tensile property, until all parameter levels have an S/N ratio for each tensile property.

The range of these S/N ratios are then computed to determine which parameter has the largest effect on each output. In addition, one-way analysis of variance (ANOVA) is done to further explore the differences between output data by printing parameters. This is done by grouping the results into different parameter levels as their factors for the one-way ANOVA. The groupings are done individually per printing parameter in order to determine how much this individual parameter affects the output tensile property.

### 2.6. External Appearance and Internal Structure Characterization

Characterization on both external appearance and internal structure of printed specimens are conducted. There are two main purposes, including to measure the geometric errors of the print and to clarify the effect of the printer parameters to the tensile properties. After the specimens have undergone the tensile tests, images of the external appearance are taken using a camera Panasonic Lumix (DMC-ZS20, Panasonic, Kadoma, Japan). A pre-preparation of the internal structure characterization is done by cutting a thin cross-sectional area from the middle of the broken specimens. These cross-sectional images are then captured to be analyzed using an Olympus SZX7 Microscope (SZX7, Olympus, Shinjuku, Japan). 

In order to quantify characterization, the cross-sectional images are then segmented using MATLAB (R2021b, MathWorks Inc., Natick, MA, USA). The segmentation is done to determine the total cross-section area in terms of square micrometers. After segmentation, the total area of the cross-section is then calculated. The segmented image is then inverted to get the area of the voids. These are then used to calculate the segmented area ratio:

(6) $$\mathrm{Void}-\mathrm{to}-\mathrm{Total}-\mathrm{Area}-\mathrm{Ratio}=\frac{\mathrm{Area}\;\mathrm{of}\;\mathrm{Voids}}{\left(\mathrm{Area}\;\mathrm{of}\;\mathrm{Voids}\right)+\left(\mathrm{Area}\;\mathrm{of}\;\mathrm{Cross}-\mathrm{section}\right)}.$$

It is then compared to the ratio of the cross-section that is based on the toolpath slicer results. This is used in order to compensate for the variances in measuring and scaling the actual area of the silicone due to the splattered cross-section. The segmented ratio error calculated would be in the equation

Error = Actual (Void–to-Total-Area-Ratio) – Ideal (Void–to-Total-Area-Ratio),(7)

This is done for all the combinations of parameters, with five specimens each. This error output would also be processed through the Taguchi analysis of mean response and S/N ratio, using the smaller-the-better formula. This is denoted by

S/N Ratio (smaller the better) = −10 × log(Σ(Y^2^)/n), (8)

## 3. Results

### 3.1. Tensile Behaviour of Silicone Printing

**Figure 4:**
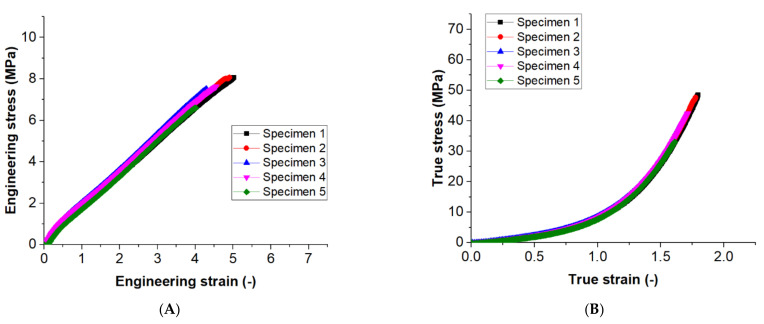
An example of (**A**) engineering stress-strain curves; and (**B**) true stress-strain curves of the combination 5 for repeatability.

Figure 4 demonstrates the experimental results of tensile behavior of silicone printing. Exampled graphs for the engineering stress-strain profile and true stress-strain profile of the tested configuration in combination 5 are shown in Figure 4A,B, respectively. Repeatability can be seen in these graphs as five curves of the individual specimen overlap. The engineering tensile strength can be observed as the maximum stress in the graph as 8.07 MPa and the average value is around 7.58 MPa. On the other hand, the true tensile strength can be observed as the maximum true stress in the graph as 48.52 MPa and the average value is around 42.3 MPa. With the effect of the shrinking cross-sectional area, the true stress increased exponentially through the uniform elongation.

**Figure 5:**
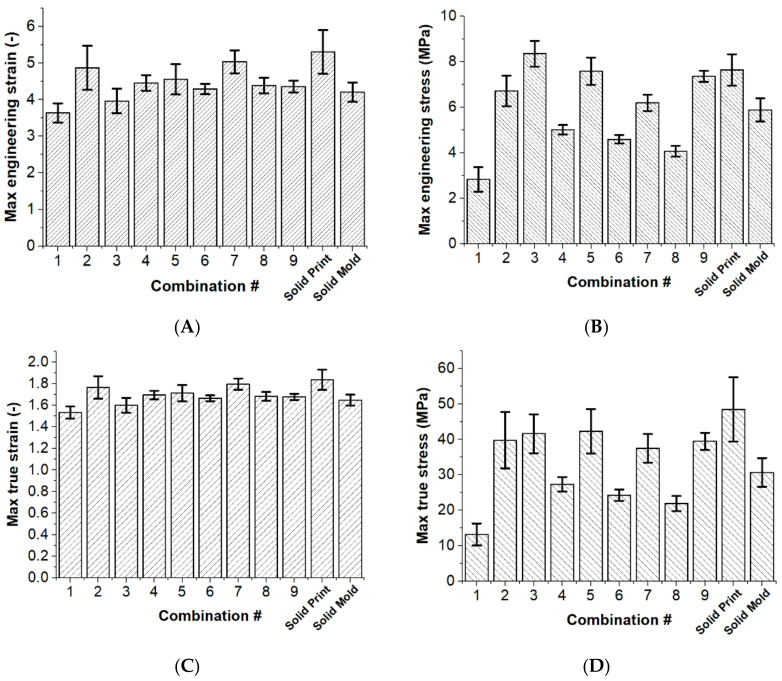
Summary of the average maximum values and standard deviations of (**A**) engineering strain; (**B**) engineering stress; (**C**) true strain; and (**D**) true stress of all combinations.

All averaged maximum values of these tensile properties are compiled in Table 2, and plotted on Figure 5A–D. These include the results of experimental matrix in the orthogonal array, and two additional cases of the 100% infilled and molded specimens. Among the combinations in the orthogonal array, the largest possible engineering strain and true strain can be found in combination 7, with a 5.03 engineering strain and a 1.80 true strain. The 100% infilled print on the other hand shows the highest value among the comparison, with a 5.3 engineering strain and a 1.84 true strain. However, the values of engineering strain and true strain of the molded specimen are smaller than many combinations, with a 4.20 engineering strain and a 1.65 true strain.

Regarding the engineering tensile strength, Figure 5B presents that combination 3 has the highest maximum tensile stress with a value of 8.35 MPa. The value is even higher than the result of the 100% infilled print specimen, which has a 7.64 MPa engineering stress. However, Figure 5D presents that the 100% infilled print specimen has the highest true stress value of 48.44 MPa instead. It can be found that consistently the lowest tensile stress value occurs in combination 1, which has the maximum value of engineering stress of 2.83 MPa and the maximum value of true stress of 13.18 MPa. 

Table 2 also shows the corresponding S/N ratios for different output responses of each combination. Based on the principle of the-higher-the-better, combination 5 shows the largest S/N ratio in the performance of strength, and combination 7 shows the largest S/N ration in the performance of strain. These indicate that combinations 5 and 7 are the most optimized settings. In contrast, combination 1 shows the least S/N ratio value in all the four tensile properties, showing it has the least optimized combination of parameters.

#### 3.1.1. Significance of Parameters—Tensile Stress

Table 3 shows the calculated S/N ratio of the tensile stress results for different levels of the individual parameters. These S/N ratio values are ranked by the ranges of variation. Three printing parameters of print speed, infill density, and flow rate show a positive increase in S/N ratio as the levels are increased. The flow rate is ranked number one with the highest engineering stress S/N ratio of 5.92 and the highest true stress S/N ratio of 6.55. This means that the level change of flow rate would contribute to the tensile property most significantly, with infill density being the second. 

**Figure 6:**
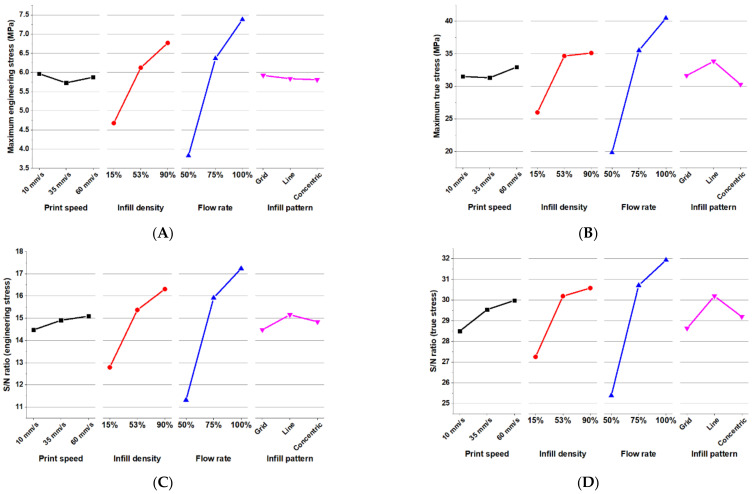
Line graphs of the effects of print parameters on the (**A**) maximum engineering stress in MPa; (**B**) maximum true stress in MPa; (**C**) engineering stress S/N ratio; and (**D**) true stress S/N ratio.

Figure 6A–D also compares the influence between the printing parameters. The trends of stress and its associated S/N ratio versus different level of individual parameters are shown to have consistent results. It can be observed that the most dramatic change in the tensile performance occurred during the increase in flow rate. Although the performance of engineering stress with regard to infill pattern is not clearly different, the infilled structure using the Line pattern presents the best performance in tensile strength. The tensile strength also shows a positive increase when the density is increased. 

#### 3.1.2. Significance of Parameters—Tensile Strain

Table 4 shows the calculated S/N ratio of the tensile strain results for different levels of the individual parameters. These S/N ratio values are ranked by the ranges of variation. The printing parameters have very little difference regarding ranges of S/N ratio values when compared to the tensile stress property. This would mean that the contribution to the elongation of each parameter is more even. The infill pattern is ranked number one with the highest engineering strain S/N ratio of 1.07 and the highest true strain S/N ratio of 0.52. 

**Figure 7:**
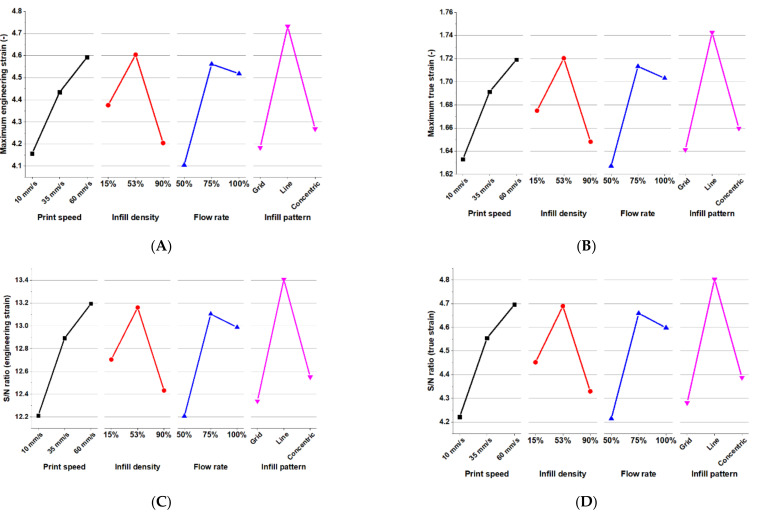
Line graphs of the effects of print parameters on the (**A**) maximum engineering strain; (**B**) maximum true strain; (**C**) engineering strain S/N ratio; and (**D**) true strain S/N ratio.

Figure 7A–D compare the influence between the printing parameters. Three parameters of infill density, flow rate, and infill pattern have the similar trends of mean tensile strain and its associated S/N ratio for different levels. Their values are increased when going from the first level to the second level, and they are decreased when going from the second to the third level. Only the parameter of print speed shows a positive relationship. It can be observed that the most dramatic change in the elongation performance occurred when using the Line infill pattern. 

#### 3.1.3. ANOVA

Table 5 summarizes the significance of parametric study using one-way ANOVA. This method indicates that the *p*-values lower than 0.05 are statistically significant. It can be seen that the variable of flow rate has a relative small *p*-value of 7.13 × 10^−12^ and 3.13 × 10^−11^ in terms of engineering stress and true stress, respectively. This means that flow rate is the most significant among all the variables at the 0.05 alpha level in terms of engineering and true stress.

### 3.2. Characterization of External Appearance

**Figure 8:**
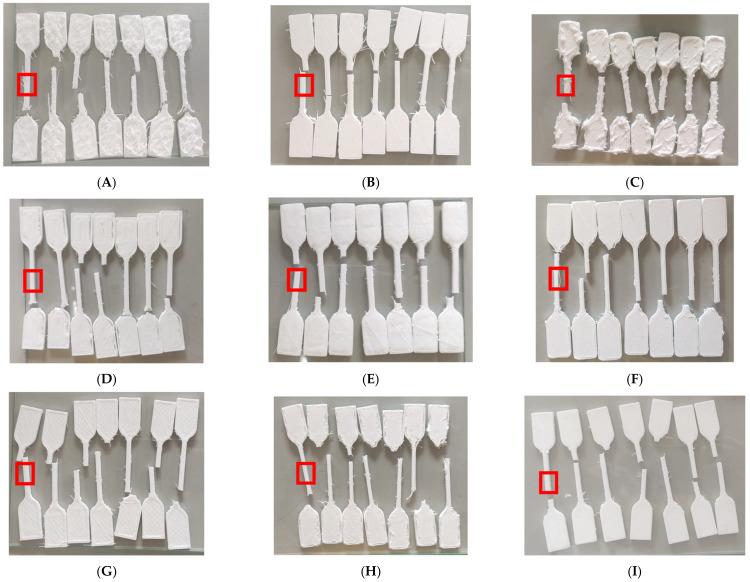
Silicone-printed specimens grouped according to their combination number as well as their printing parameter values. The red boxes indicated where the sliced cross-section is located. (**A**) Combination 1: Print speed: 10 mm/s; Infill density: 15%; Flow rate: 50%; Infill pattern: Grid. (**B**) Combination 2: Print speed: 10 mm/s; Infill density: 53%; Flow rate: 75%; Infill pattern: Line. (**C**) Combination 3: Print speed: 10 mm/s; Infill density: 90%; Flow rate: 100%; Infill pattern: Concentric. (**D**) Combination 4: Print speed: 35 mm/s; Infill density: 15%; Flow rate: 75%; Infill pattern: Concentric. (**E**) Combination 5: Print speed: 35 mm/s; Infill density: 53%; Flow rate: 100%; Infill pattern: Grid. (**F**) Combination 6: Print speed: 35 mm/s; Infill density: 90%; Flow rate: 50%; Infill pattern: Line. (**G**) Combination 7: Print speed: 60 mm/s; Infill density: 15%; Flow rate: 100%; Infill pattern: Line. (**H**) Combination 8: Print speed: 60 mm/s; Infill density: 53%; Flow rate: 50%; Infill pattern: Concentric. (**I**) Combination 9: Print speed: 60 mm/s; Infill density: 90%; Flow rate: 75%; Infill pattern: Grid.

The external appearance of the silicone-printed specimens grouped by different configurations can be seen in Figure 8. The remains of stringing are immediately noticeable. In the top surfaces of Figure 8A,D,F,H contain holes that expose its inner structure. In contrast, Figure 8B,C,E,G,I show a solid top surface. 

### 3.3. Characterization of Internal Structure

**Figure 9:**
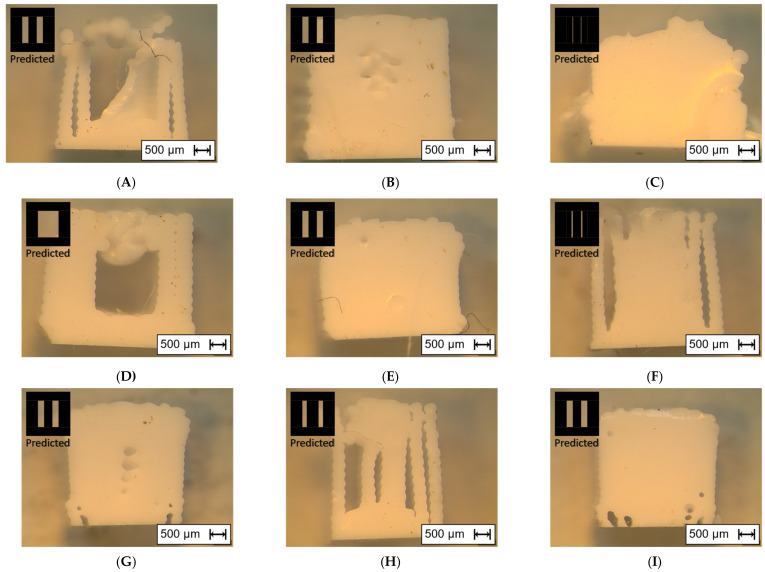
Cross-sectional images captured by Olympus SZX7, their ideal results, and their corresponding printing parameters. (**A**) Combination 1: Print speed: 10 mm/s; Infill density: 15%; Flow rate: 50%; Infill Pattern: Grid. (**B**) Combination 2: Print speed: 10 mm/s; Infill density: 53%; Flow rate: 75%; Infill pattern: Line. (**C**) Combination 3: Print speed: 10 mm/s; Infill density: 90%; Flow rate: 100%; Infill pattern: Concentric. (**D**) Combination 4: Print speed: 35 mm/s; Infill density: 15%; Flow rate: 75%; Infill pattern: Concentric. (**E**) Combination 5: Print speed: 35 mm/s; Infill density: 53%; Flow rate: 100%; Infill pattern: Grid. (**F**) Combination 6: Print speed: 35 mm/s; Infill density: 90%; Flow rate: 50%; Infill pattern: Line. (**G**) Combination 7: Print speed: 60 mm/s; Infill density: 15%; Flow rate: 100%; Infill pattern: Line. (**H**) Combination 8: Print speed: 60 mm/s; Infill density: 53%; Flow rate: 50%; Infill pattern: Concentric. (**I**) Combination 9: Print speed: 60 mm/s; Infill density: 90%; Flow rate: 75%; Infill pattern: Grid.

In Figure 8, the red boxes are used to indicate the location of cut for the cross-section characterization. The corresponding characterizations for each combination can be seen in Figure 9. On the left-top corner of these images, the ideal shape of the cross-sectional patterns is present. It can be clearly observed in micro-scale that the internal structure does not match the ideal shapes. This would be attributed to deviations in locations of cutting. 

In the results, the staircase effect is mainly observed at the sides. Regarding the internal structures, Figure 9A shows a broken phenomenon and a collapsed top surface, similar to Figure 9D. Figure 9B,D,E,G,I have fused internal structures with some voids within the solid surface. In Figure 9F,H, the internal structures are shown to have vertically sliced features.

### 3.4. Quantitative Analysis of Image Segmentation

**Figure 10:**
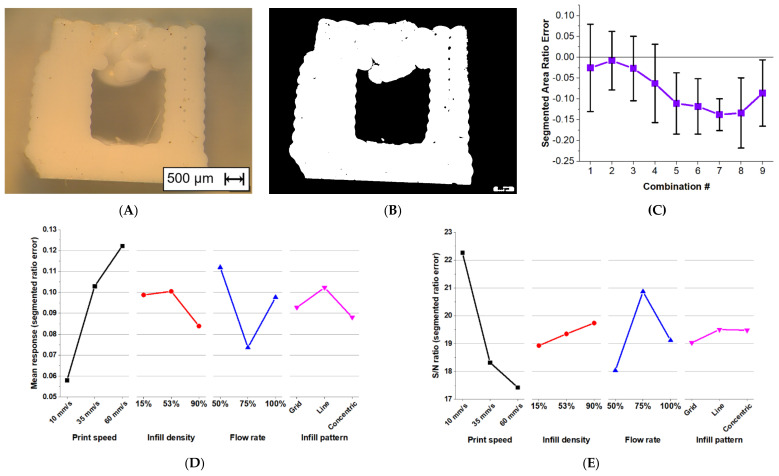
(**A**) Cross-sectional images captured by Olympus SZX7; (**B**) image process via MATLAB; (**C**) the resulting segmented area ratio error for each combination. Line graphs of the effects of print parameters on the segment ratio error in terms of (**D**) mean response, and (**E**) S/N ratio response.

For a quantitative study of the geometric errors in printing, an example cross-section image and its resulting segmentation can be seen in Figure 10A,B, respectively. The segmented ratio error results for all combinations are shown in the line graph of Figure 10C. This graph shows that the mean values for each output are all below zero, showing that the ratio from the actual result would be smaller. The closest combination to a value of zero is combination 2, with a value of 0.008. The graphs of mean response and S/N ratio are shown in Figure 10D,E. Their S/N response values are then displayed on Table 6, where it is ranked according to their ranges. 

It can be observed in Figure 10D that the segmented ratio error increases in value when increasing the print speed from 10 mm/s to 60 mm/s. The lowest mean response would then be 0.06 in the 10 mm/s level. This means that the ratio of voids to the total area is preserved due to the slow print speed of depositing the silicone. The S/N ratio also shows that less variability can be seen in the lower print speed level. This decreasing S/N ratio as the level of print speed increases shows that the variability increases with increasing print speed. The print speed also shows the largest range in all parameters, having an S/N response range of 4.84.

Figure 10D also shows that an increase in the level of infill density decreases the value of segment ratio error, and the lowest mean response is the value of 0.08. The S/N ratio graph on the other hand increases in value for increasing infill density level, with the highest S/N response value of 19.74. This shows less error and variability in the larger infill density. 

## 4. Discussion

### 4.1. Parametric Studies for Tensile Properties

As stated in the results of tensile stress, the flow rate has shown the highest rank in terms of S/N ratio and is the one with the most significance in ANOVA. The reason is that in having higher flow rate, more material would thus be deposited onto the cross-sectional area of the dumbbell specimens. A larger cross-sectional area would thus mean that the dumbbell specimen could withstand more forces, as these forces are distributed in a larger area. The other reason that flow rate would have a higher contribution than infill density would be that the structure is small enough that the outer shells accommodate a larger portion of the cross-sectional area. Structures with larger cross-sectional area would then be more dependent on the infill density in terms of tensile stress as compared to the flow rate. The optimal parameters found for engineering and true stresses are 60 mm/s print speed, 53% infill density, 100% flow rate, and line infill pattern.

Regarding the behavior of tensile strain, the studied parameters have very close values in terms of S/N ratios, reflecting that the performance depends on the printed structure as a whole rather than relying on one specific parameter. The infill density and flow rate has consistently shown a large range in the S/N ratios for the two tensile properties. Thus, these two would be the first parameters that need consideration in order to get the higher tensile properties when designing. For a maximum tensile strain, the optimal printing parameters are the 53% infill density with a 75% flow rate. On the other hand, an optimal printing parameter for the maximum tensile stress is achieved with a 90% infill density with a 100% flow rate. For both tensile properties, the most optimal condition of print speed is 60 mm/s, and for the infill pattern it is the Line pattern.

Although the tensile strength seems highly correlated to the density of specimens, this study demonstrates that the result of a solid specimen density does not always present the highest engineering tensile strength and the longest strain. In Table 2, the maximum engineering stress of the molded specimen presents only 5.88 MPa, which is only two-thirds of the best condition in this study. The molded specimen results may be attributed to the difficulty in pouring the mold due to its viscosity, however the element structure and reactions of the particles are not discussed in this study. It is believed that the two solid specimens manufactured by using a 100% infilled printing and molding present very different results. This shows that using the printer is a more reliable way to fabricate silicone structures with better tensile properties. 

In addition, comparing the experimental results of the tensile stress to the specification given by the company supplier, it is shown that the lowest value of true stress (13.18 MPa) in this study exceeds the specification of 12.07 MPa. With the assistance of parametric study, applying the optimal parameter configuration in printing has the potential to improve tensile performance.

### 4.2. Parametric Studies for External Appearance

The remaining strings on the external appearance can be seen in all the printed specimens in this study. This is due to the mechanism of a Bowden-type silicone printer but lacking retraction due to the difficulty in controlling the flow of gel-like materials. Such a silicone printing requires post processing for the string removal after the specimens are cured. This issue of clean appearance does not affect the tensile properties, but it may cause other phenomena such as holes, voids, and oozes.

Figure 8A,F,H show holes in the structures as stated in the results. These resulting prints with low flow rate levels mean that their structures are not fused together properly, leading to the low performance of their corresponding combinations in terms of both tensile stresses seen in the graphs of Figure 5B,D. Figure 8D also exhibits holes in its structure, but this may also be due to the infill pattern not providing enough foundation for the top surface layer. In contrast, Figure 8B,C,E,G,I all show a solid top surface, which is reflected in their corresponding combinations having larger tensile stresses in the graphs of Figure 5B,D.

Having a very low printing speed on the other hand accentuates the extreme levels of the flow rates. In Figure 8A, the speed is low, and the flow rate is high that there is almost no infill pattern formed and a top surface is missing in the structure. This leads to poor performance in both tensile properties of combination 1. In contrast, Figure 8C with its large flow rate and low print speed formed ooze, distorting the dumbbell specimen. It thus leads to combination 3 having a larger than usual engineering stress in Figure 5B,D when compared to combination 2. Figure 8B is the cross-section of combination 2, which has the same speed as combination 3 but smaller flow rate, showing no ooze. The low print speed with the two different flow rates is the only one that shows visually prominent effects in the combinations. Figure 8B, as well as those with higher print speeds all show consistent dumbbell shapes that have solid top surfaces with minimal oozing.

Results of external structures with lower flow rates show poor formation of top surfaces. Lower print speeds accentuate the extreme values in flow rate and exhibits phenomena of oozing in addition to the poor formation of the top surface. Internal characterization of cross-sections results shows infill densities of 53% and 90%, and exhibits solid cross-sections due to the pre-cured silicone’s liquid behavior. Low flow rate combinations exhibited poor layer fusion. Line infill patterns shows its consistency and advantage in the design of its patterns to exhibit high values and influence in the tensile properties. Quantitative area segmentation results show that print speeds and flow rate have the most contribution to this output, with slower print speeds showing less error, as well as the 75% flow rate.

### 4.3. Parameter Effects on Internal Structure

The parameter effects on the internal structure can be referred to the results of the cross-section images in Figure 9. The higher infill densities demonstrate more solid cross-sectional images. Figure 9A shows a hollow and collapsing top surface due to the lowest infill density of 15%. It is an objective to avoid collapsing top surfaces and internal voids due to the gel-like nature of pre-cured silicone. Otherwise, this consequence affects the tensile properties. The solid images of Figure 9E,I proves the higher values of the corresponding combinations in terms of tensile stress in Figure 5B,D. Even if the infill density is only 90%, the cross-sectional images already have no visible voids of any kind in Figure 9C,I.

Infill pattern seems to be not the only parameter that affects the amount of void. For example, the resulting cross-sections look mostly solid in Figure 9E,I. Compared to Figure 9A, the results are hollow even with the same Grid infill pattern as Figure 9E,I. This difference can thus be attributed to the other parameters such as the higher flow rates and higher densities. For the line infill pattern, it consistently shows that it is mostly solid even when the infill density is in the low value of 15%, seen in Figure 9G. On the other hand, Figure 9C shows a splattered cross-sectional result with the concentric infill pattern. This suggested that the slower print speed with a higher flow rate and a higher infill density deposited more silicone than intended. 

It is difficult to distinguish the effect of flow rate on the amount of voids. An increase from 50% to 75% of flow rate shows a decrease in segment ratio error value, but the increase from 75% to 100% flow rate shows an increase instead. Figure 10E shows that an increase from 50% to 75% flow rate results in an increase in S/N ratio, and the opposite happens when increasing from 75% to 100% flow rate. Compared to the infill density, the flow rate shows variation.

In silicone printing, the resulting staircase effect on the appearance seems correlated with lower flow rates. When increasing the flow rate, influence of the staircase effect on the resulting print decreases via the observation from the microstructure of the cross-sectional images. The layers in the cross-section of the printed specimens look fused in appearance. However, the tensile tests of ASTM D412 is uniaxial, along the length of the dumbbell specimen. The tensile strength influenced by the staircase effect when printing on the other orientation can be discussed in a future study.

### 4.4. Comparison with Earlier Studies

One study that also made use of tensile testing to discuss silicone printing results would be [6], where they also related void properties in addition to tensile testing. They varied the parameters of compression factor, number of outline, and infill angle for their experiments. From their results, they also found that their baseline, a sheet stamped specimen, observed the highest tensile stress of 1.63 MPa and their fabricated specimens in the 1.44 MPa to 1.55 MPa range. Their resulting tensile strain ranges from 6.0 to 7.1, with an average of 6.5. Their tensile stress results are lower than the results found in this study as the specimen they used was thinner, having a thickness of 0.2 mm compared to this study’s 3 mm. This low thickness also explains the larger tensile strain range, even when compared to the engineering strains of this study. They concluded that void size has a significant impact in tensile stress. This can also be seen in the findings of this paper, through looking at Figure 5, combinations 3, 5, and 9 exhibited the highest tensile stress in the orthogonal array experiments, and it would be seen that in Figure 9, they also exhibited the minimum voids.

Although the focus of the study in [4] is to create an optimization strategy in 3D printing soft materials using FRE, they also conducted tensile testing to support their results. From their representative curve, their polydimethylsiloxane (PDMS) behaved in a linearly elastic behavior until failure. This is also observed in the engineering stress-strain curve in Figure 5 of this study. They have lower values in tensile properties, with a maximum tensile stress of around 2 MPa and a tensile strain of around 1.5, stemming from the difference in PDMS and silicone rubber.

Similar trends in parameter studies can be observed in Taguchi studies in [2,3,23], but using the typical PLA for extruder material. The study in [2] also observed infill pattern and infill density parameters. They found a similar result of infill density being the highest influence on the specimen’s Young’s modulus, yield strength, and tensile strength, and that increasing infill density would also increase the mean response and S/N ratio response of those output parameters. This significance of infill density to the tensile strength can also be confirmed in [3], where it also has the highest influence and increasing the percentage infill also increases the tensile strength to weight ratio. This study also observed the influence of print speed, but similar to what is observed in this study, it has less influence as compared to infill density. Along with the infill density, the flow rate is another parameter studied in [23]. The same behavior of infill density and its significance is also observed in this study, but this time having larger significance than flow rate, contrary to what is found from Figure 6 in this study. This difference can be attributed to the fluidic nature of the silicone material extruded.

## 5. Conclusions

This paper presents the relationship of different parameter levels toward the tensile properties of silicone dumbbell specimens printed using a Bowden-type silicone printer. The experimental investigations are based on the Taguchi method. Images characterization of external appearance and internal structure of printing provide the evidence of parameter effects. The results suggest that for the engineering and true tensile stress, the changes in flow rate have the most significant influence from both mean response and S/N ratio graphs, followed by changes in infill density. For the engineering and true strain, the contributions of the printing parameters have more equal ranges, with the infill pattern the one with the highest influence. The above trends of the parametric studies can serve as the guideline for printing three-dimensional silicone structures. The contribution of this study can increase the feasibility of printing biocompatible and multifunctional silicone material and further widen the accessibility and customizability of silicone for medical applications.

## Figures and Tables

**Table 1:** A table of experimental configuration of the Taguchi L9 Orthogonal Array.

Combination Number	Print Speed (mm/s)	Infill Density (%)	Flow Rate (%)	Infill Pattern (-)
1	10	15	50	Grid
2	10	53	75	Line
3	10	90	100	Concentric
4	35	15	75	Concentric
5	35	53	100	Grid
6	35	90	50	Line
7	60	15	100	Line
8	60	53	50	Concentric
9	60	90	75	Grid

**Table 2:** A table of the corresponding means, standard deviations (SD), and S/N ratios for each tensile property of each combination.

Combination Numbers	Tensile Properties								S/N Ratio			
	ε_e_ (-)		σ_e_ (MPa)		ε_t_ (-)		σ_t_ (MPa)		ε_e_ (dB)	σ_e_ (dB)	ε_t_ (dB)	σ_t_ (dB)
	Mean	SD	Mean	SD	Mean	SD	Mean	SD				
1	3.64	0.26	2.83	0.54	1.53	0.06	13.18	3.11	11.16	8.59	3.70	21.75
2	4.87	0.60	6.72	0.67	1.77	0.10	39.75	7.91	13.59	16.44	4.90	31.57
3	3.96	0.34	**8.35**	0.57	1.60	0.07	**41.60**	5.46	11.88	**18.39**	4.06	32.19
4	4.46	0.21	5.01	0.21	1.70	0.04	27.34	2.06	12.95	13.98	4.58	28.67
5	4.56	0.42	7.58	0.60	1.71	0.08	42.30	6.29	13.08	17.52	4.65	**32.27**
6	4.29	0.14	4.59	0.19	1.67	0.03	24.31	1.58	12.64	13.22	4.43	27.67
7	**5.03**	0.31	6.20	0.36	**1.80**	0.05	37.48	4.03	**13.99**	15.81	5.08	31.34
8	4.39	0.22	4.07	0.24	1.68	0.04	21.93	2.09	12.81	12.15	4.52	26.73
9	4.36	0.16	7.36	0.24	1.68	0.03	39.46	2.44	12.77	17.32	4.49	31.88
100% Infilled	**5.30**	0.60	**7.64**	0.69	**1.84**	0.09	**48.44**	9.07	11.16	13.59	11.88	12.95
Molded	4.20	0.26	5.88	0.51	1.65	0.05	30.69	4.06	13.08	12.64	**13.99**	12.81

**Table 3:** Output tensile stress S/N ratio response per parameter level.

	Print Speed	Infill Density	Flow Rate	Infill Pattern
Max Engineering Stress				
Level 1	14.47	12.79	11.32	14.48
Level 2	14.91	15.37	15.92	15.16
Level 3	15.09	16.31	17.24	14.84
Range	0.62	3.52	**5.92**	0.68
Rank	4	2	**1**	3
Max True Stress				
Level 1	28.50	27.25	25.38	28.63
Level 2	29.54	30.19	30.71	30.19
Level 3	29.98	30.58	31.93	29.20
Range	1.48	3.33	**6.55**	1.56
Rank	4	2	**1**	3

**Table 4:** Output tensile strain S/N ratio response per parameter level.

	Print Speed	Infill Density	Flow Rate	Infill Pattern
Max Engineering Strain				
Level 1	12.21	12.70	12.21	12.34
Level 2	12.89	13.16	13.10	13.41
Level 3	13.19	12.43	12.99	12.55
Range	0.98	0.73	0.90	**1.07**
Rank	2	4	3	**1**
Max True Strain				
Level 1	4.22	4.45	4.21	4.28
Level 2	4.55	4.69	4.66	4.80
Level 3	4.70	4.33	4.60	4.39
Range	0.48	0.36	0.44	**0.52**
Rank	2	4	3	**1**

**Table 5:** Summary of ANOVA Results.

	Print Speed		Infill Density		Flow Rate		Infill Pattern	
	F	P	F	P	F	P	F	P
Engineering strain	3.21	0.05 *	2.58	0.09	4.40	0.018 *	6.55	0.0033 *
Engineering stress	0.06	0.94	6.69	0.0030 *	50.29	7.13 × 10^−12^ *	0.02	0.98
True strain	3.80	0.030 *	2.47	0.10	4.44	0.018 *	6.28	0.0041 *
True stress	0.10	0.91	3.86	0.029 *	45.44	3.13 × 10^−11^ *	0.41	0.67

* Significant at the 0.05 alpha level.

**Table 6:** Output segmented ratio error S/N ratio response per parameter level.

	Print Speed	Infill Density	Flow Rate	Infill Pattern
Segment ratio error				
Level 1	22.27	18.93	18.04	19.03
Level 2	18.32	19.35	20.87	19.51
Level 3	17.43	19.74	19.11	19.48
Range	**4.83**	0.81	2.83	0.47
Rank	**1**	3	2	4

## Data Availability

Not applicable.
